# Modeling Retinal Degeneration Using Patient-Specific Induced Pluripotent Stem Cells

**DOI:** 10.1371/journal.pone.0017084

**Published:** 2011-02-10

**Authors:** Zi-Bing Jin, Satoshi Okamoto, Fumitaka Osakada, Kohei Homma, Juthaporn Assawachananont, Yasuhiko Hirami, Takeshi Iwata, Masayo Takahashi

**Affiliations:** 1 Laboratory for Retinal Regeneration, RIKEN Center for Developmental Biology, Kobe, Japan; 2 School of Optometry and Ophthalmology, Eye Hospital, Wenzhou Medical College, Wenzhou, China; 3 Systems Neurobiology Laboratory, The Salk Institute for Biological Studies, La Jolla, California, United States of America; 4 National Institute of Sensory Organs, National Hospital Organization Tokyo Medical Center, Tokyo, Japan; 5 Center for iPS Research and Application, Kyoto University, Kyoto, Japan; National Institute on Aging Intramural Research Program, United States of America

## Abstract

Retinitis pigmentosa (RP) is the most common inherited human eye disease resulting in night blindness and visual defects. It is well known that the disease is caused by rod photoreceptor degeneration; however, it remains incurable, due to the unavailability of disease-specific human photoreceptor cells for use in mechanistic studies and drug screening. We obtained fibroblast cells from five RP patients with distinct mutations in the *RP1*, *RP9*, *PRPH2* or *RHO* gene, and generated patient-specific induced pluripotent stem (iPS) cells by ectopic expression of four key reprogramming factors. We differentiated the iPS cells into rod photoreceptor cells, which had been lost in the patients, and found that they exhibited suitable immunocytochemical features and electrophysiological properties. Interestingly, the number of the patient-derived rod cells with distinct mutations decreased *in vitro*; cells derived from patients with a specific mutation expressed markers for oxidation or endoplasmic reticulum stress, and exhibited different responses to vitamin E than had been observed in clinical trials. Overall, patient-derived rod cells recapitulated the disease phenotype and expressed markers of cellular stresses. Our results demonstrate that the use of patient-derived iPS cells will help to elucidate the pathogenic mechanisms caused by genetic mutations in RP.

## Introduction

Retinitis pigmentosa (RP) leads inevitably to visual impairment due to irreversible retinal degeneration, specifically of primary rod photoreceptors. The condition causes night blindness and visual field defects. The disease onset spans a wide range of ages, but RP most often occurs in late life. There is no treatment that allows patients to avoid deterioration of visual function. RP encompasses a number of genetic subtypes, with more than 45 causative genes and a large number of mutations identified thus far. The genetic heterogeneity of RP suggests a diversity of disease mechanisms, which remain largely unclear. Furthermore, for many of the RP subtypes, no appropriate animal models are available. Although large clinical trials have been conducted with α-tocopherol and β-carotene, these studies found no statistically significant change of visual function in RP patients [1], [2]. The underlying mutations causing disease in the patients tested in the clinical trials were not revealed, and the variability of individual responses to these drugs is unknown. One of the reasons why these clinical trials failed to examine the effectiveness of drugs is that the effect of a drug may be different between patients with different underlying mutations.

Induced pluripotent stem (iPS) cells reprogrammed from somatic cells [3], [4] have enabled us to easily generate patient-derived terminally differentiated cells *in vitro* [5]–[7]. We have successfully induced differentiation of photoreceptor cells from both human embryonic stem (ES) cells [8] and iPS cells [9], [10]. Modeling pathogenesis and treatment *in vitro* using patient iPS cell-derived photoreceptors will elucidate disease mechanisms; circumvent problems related to differences among species that arise when using animal models; decrease patient risk; and reduce the cost of early-stage clinical trials. Here, we generated iPS cells from RP patients with different mutations and demonstrated the potential of patient-derived photoreceptors for disease modeling.

## Materials and Methods

### RP patients and genetic mutations

**Figure 1:**
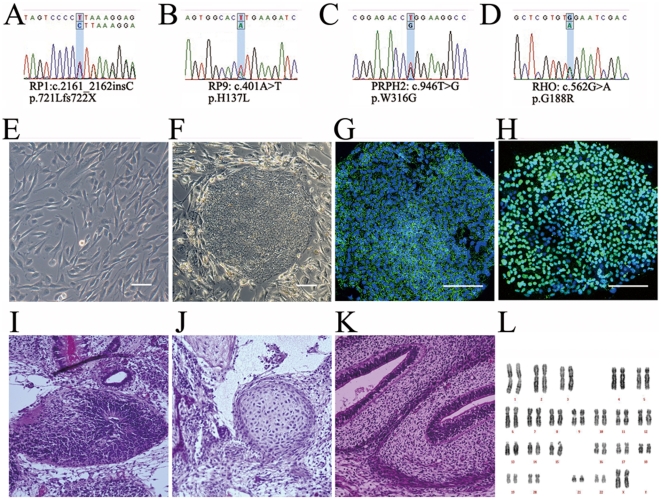
iPS cells derived from RP patients. Mutations identified in patients K21 (RP1) (**A**), K11 and K10 (RP9) (**B**), P101 (PRPH2) (**C**), and P59 (RHO) (**D**). Patient-derived fibroblast cells (**E**) were reprogrammed into iPS cells (**F**). The iPS cells expressed SSEA-4 (**G**) and Nanog (**H**). A teratoma formation test confirmed iPS cells' ability to generate all three germ layers: endoderm (**I**), mesoderm (**J**) and ectoderm (**K**). Karyotype analysis (**I**). Scale bars, 50 µm.

The protocol of this study adhered to the tenets of the Declaration of Helsinki. The study was approved by the ethical committees of the Institute of Biomedical Research and Innovation Hospital and the RIKEN Center for Developmental Biology, Japan. Written informed consent from all patients was obtained. We selected five RP patients from four families whose disease-causing mutations have been identified (**Fig. 1A–D** **and Fig. S1**). Of the five RP patients in this study, three late-onset patients carried the following mutations: 721Lfs722X in *RP1*, W316G in *PRPH2*, and G188R in *RHO*. Two relatively early-onset patients from the same family carried a H137L mutation in *RP9*, which we confirmed by both genomic and cDNA sequencing (**Fig. S2**). All patients showed typical manifestations of RP (**Tab. S1**). Peripheral blood obtained from patients was used for DNA isolation. A comprehensive screening of disease-causing genes was carried out as described previously [11]. For the RP9 mutation, total RNA was isolated from fresh blood samples and iPS cells, and synthesized cDNA was subjected to PCR and direct sequencing to confirm whether the mutation was located in the *RP9* gene or the pseudo-RP9 gene (paralogous variant). Both fibroblast and iPS cells were analyzed to re-confirm the identified mutation.

### iPS cells generation

To generate iPS cells, retroviral transduction of Oct3/4, Sox2, Klf4, and c-Myc into patient-derived fibroblast cells was carried out as described previously [3]. Established iPS cell lines were maintained on a feeder layer of mitomycin C-treated SNL cells (a murine-derived fibroblast STO cell line expressing the neomycin-resistance gene cassette and LIF) in a humidified atmosphere of 5% CO_2_ and 95% air at 37°C. Cells were maintained in DMEM-F12 supplemented with 0.1 mM non-essential amino acids, 0.1 mM 2-mercaptoethanol, 2 mM L-glutamine, 20% KnockOut Serum Replacement (KSR), and 4 ng/ml basic fibroblast growth factor (Upstate Biotechnology).

### Transgene quantification

To examine the copy number of transgenes integrated into the host genome, DNA was isolated and quantitative detection of viral transgenes was performed using real-time PCR. The endogenous gene was used as a control. Before quantitative PCR, a standard curve for each primer and/or probe set was determined using a set of plasmid DNA dilutions. Taqman qPCR to detect integrated OCT3/4, KLF4, and MYC was performed using 20 µl reactions consisting of 10 µl TaqMan Master Mix with uracil N-glycosylase, 4.9 µM primers, 250 nM probe, and 1 µl of the DNA sample. Quantification of viral SOX2 was assayed using SYBR Green.

### Teratoma formation

Animal protocols were approved by the RIKEN Center for Developmental Biology ethical committee (No. AH18-05). A total of 10^7^ trypsinized iPS cells were injected subcapsularly into the testis of SCID mice (two mice per iPS cell line). Four weeks later, the testis was fixed and sectioned for H&E staining.

### Immunocytochemistry

Cells were fixed with 4% paraformaldehyde for 15 min at 4°C and then permeabilized with 0.3% Triton X-100 for 45 min. After 1 h blocking with 5% goat serum, cells were incubated with primary antibodies overnight at 4°C and subsequently with secondary antibodies for 1 h at room temperature. The primary and second antibodies used are listed in **Tab. S2**.

### Karyotype analysis

Karyotype analysis of the iPS cell chromosomes was carried out using a standard G-band technique (300–400 band level).

### Photoreceptor differentiation and drug testing

*In vitro* differentiation of rod photoreceptor cells was performed as previously reported [8], but with a minor modification. To find a KSR optimal for retinal differentiation, lot testing was conducted before differentiation. iPS colonies were dissociated into clumps with 0.25% trypsin and 0.1 mg/ml collagenase IV in PBS containing 1 mM CaCl_2_ and 20% KSR. Feeder cells were removed by incubation of the iPS cell suspension on a gelatin-coated dish for 1 h. iPS clumps were moved to a non-adhesive MPC-treated dish (NUNC) in maintenance medium for 3 days, in 20% KSR-containing differentiation medium (DMEM-12 supplemented with 0.1 mM non-essential amino acids, 0.1 mM 2-mercaptoethanol, 2 mM L-glutamine) for 3 days, then in 15% KSR-containing differentiation medium for 9 days, and finally in 10% KSR-containing medium for 6 days. Cells were treated with Lefty-A and Dkk-1 during floating culture. At day 21, the cells were plated en bloc on poly-D-lysine/laminin/fibronectin−coated 8-well culture slides (BD Biocoat) at a density of 15–20 aggregates/cm^2^. The cells were cultured in 10% KSR-containing differentiation medium until day 60. Cells were further treated with 100 nM retinoic acid (Sigma) and 100 µM taurine (Sigma) in photoreceptor differentiation medium (GMEM, 5% KSR, 0.1 mM non-essential amino acids, 0.1 mM 2-mercaptoethanol, 1 mM pyruvate, N2 supplement, and 50 units/ml penicillin, 20 µg/ml streptomycin). Differentiated cells from both normal and patient iPS cells were treated with 100 µM α-tocopherol, 200 µM ascorbic acid and 1.6 µM β-carotene starting at differentiation day 120. One week later, cells were fixed for immunostaining.

### Electrophysiological recording

Recombinant lentiviral vectors expressing GFP under the control of the Nrl or RHO promoters were generated in HEK293t cells (RIKEN Cell Bank), and differentiated cells were infected with virus on day 90. Cells expressing GFP were targeted for patch clamp recordings. Voltage-clamp recordings were performed with 12–15 MΩ glass electrodes. Signals were amplified using Multiclamp 700B amplifiers (Molecular Devices). The internal solution was 135 mM K-gluconate, 10 mM HEPES, 3 mM KCl, 0.2 mM EGTA, 2.5 mM MgCl_2_, 5 mM adenosine 5′-triphosphate, 0.3 mM guanosine-5′-triphosphate, 0.06 mM Alexa Fluor 594 (Molecular probes), adjusted to pH 7.6 with KOH. The retinal cells were perfused with oxygen-bubbled external medium: 23 mM NaHCO_3_, 0.5 mM KH_2_PO_4_, 120 mM NaCl, 3.1 mM KCl, 6 mM Glucose, 1 mM MgSO_4_, 2 mM CaCl_2_, and 0.004% Phenol red. The medium was heated to 37°C with a temperature controller (Warner Instruments).

### Cell count and statistical analysis

Differentiated cells visualized with specific antibodies were counted blindly by an independent observer. Data are expressed as means ± s.e.m. The statistical significance of differences was determined by one-way ANOVA followed by Tukey's test or Dunnett's test, or by two-way ANOVA followed by Bonferroni test using the GraphPad Prizm software. Probability values less than 0.05 were considered significant.

## Results

### Generation of iPS cell lines from patients with RP

Mutations identified in the five patients were confirmed by bi-directional sequencing (**Fig. S1**). Through genotyping of four patients and two normal relatives in the RP9 family, we found the H137L mutation in the *RP9* gene co-segregated with the disease, strongly indicating that the mutation is indeed the genetic cause of the disease. We cultured fibroblasts from skin samples of these patients on gelatin-coated dishes (**Fig. 1E**) and infected them with retroviral vectors encoding *OCT3/4* (also known as *POU5F1*), *SOX2*, *KLF4*, and *c-MYC*, using a previously established method [3]. Each mutation was re-confirmed in both fibroblasts and iPS cells. Established iPS colonies showed human embryonic stem cell-like morphology (**Fig. 1F** **and Fig. S3A**) and expressed pluripotency markers (**Fig. 1C–D**). We selected iPS cell lines for each patient using multiple criteria. First, we excluded iPS cell lines in which spontaneous differentiation occurred repeatedly during maintenance (**Fig. S3B**). We chose iPS colonies that maintained morphologies similar to those of human ES cells through more than 10 passages. Second, we quantified the transgene copy number and selected iPS cell lines with the fewest integrations, as the risk of gene disruption through random insertion increases with the number of transgenes (**Fig. S4A–E**). Third, in order to select iPS cell lines with full pluripotency, we verified the ability to form teratomas. Teratomas formed by injecting iPS colonies into the testis *in vivo* showed contributions to all three embryonic germ layers: ectoderm, mesoderm, and endoderm (**Fig. 1E–G**). Finally, karyotype analysis was carried out to examine the chromosome integrity. The patient-iPS cells showed normal karyotypes after extended passage, indicating chromosomal stability (**Fig. 1H**). These results provide *in vitro* and *in vivo* functional proof of pluripotency for RP patient-derived iPS cells.

### Generation of patient-specific retinal photoreceptor

**Figure 2:**
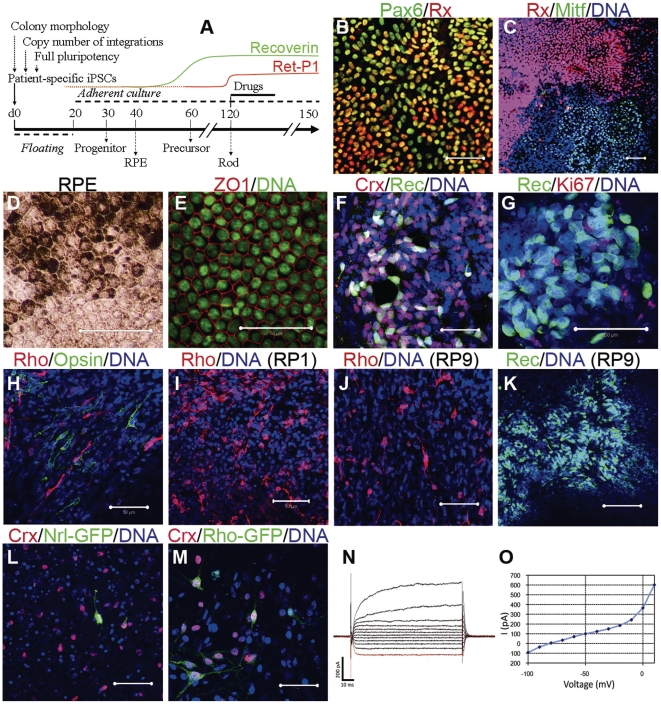
Induction of patient-specific retinal photoreceptor cells. Retinal cells were induced sequentially by *in vitro* differentiation. (**A**) Experimental schema. (**B**) Neural retina progenitor cells (Pax6+Rx+) and RPE progenitor cells (Mitf+) were separated in the culture dish (**C**). Patient-specific RPE cells exhibited hexagonal morphology and pigmentation (**D**) and expressed the tight junction marker ZO-1 (**E**). Photoreceptor cells were positive for immature photoreceptor markers Crx and Recoverin on day 60 (**F**). Recoverin+ cells did not co-express Ki67, a proliferating cell marker (**G**). Differentiation of rod photoreceptors (Rhodopsin+) and cone photoreceptors (Opsin+) from patient iPS cells (**H**). Rhodopsin + rod photoreceptors induced from K21-iPS at day 120 (**I**). K11-derived rod photoreceptors were observed at day 120 (**J**). No Rhodopsin+ cells were detected, but Recoverin+ cells were present at day 150(**K**). Induced rod photoreceptor cells (Crx+) labeled with lentiviral vectors encoding GFP driven by a rod photoreceptor-specific promoter Nrl (**L**: Nrl-GFP) or Rhodopsin (**M**: Rho-GFP). Arrows indicate cells co-expressing Crx and GFP. (**N**) Whole-cell recording of rod photoreceptor cell differentiated human iPS cells. Recorded cells expressed GFP under the control of the Rhodopsin promoter. (**O**) Relationship between voltage and membrane current (i) produced a non-linear curve, suggesting that voltage-dependent channels exist in iPS cell-derived rod photoreceptors Rec, Recoverin; Rho, Rhodopsin. Scale bars, 50 µm.

We previously demonstrated *in vitro* differentiation of retinal photoreceptor cells from wild-type human ES [8] and iPS cells [9], [10] using a stepwise differentiation method known as serum-free culture of embryoid body-like aggregates [12]. We first evaluated the differentiation efficiency of three selected iPS cell lines of the five patients (**Fig. 2A**). Retinal progenitor, photoreceptor precursor, retinal pigment epithelium (RPE) and rod photoreceptor cells were sequentially induced (**Fig. 2B–K**), consistent with our previous studies [8]–[10], [12]. All patient-derived iPS cell lines differentiated into RPE cells that form ZO-1+ tight junctions on differentiation day 60, with timing, morphology, and efficiency similar to that of wild-type iPS cells (**Fig. 2D–E** **; Fig. S5**). Immature photoreceptors expressing Crx and Recoverin (day ∼60) were observed as clusters in the colonies (**Fig. S6A–B**). The patient-iPS cells also differentiated into blue Opsin+ or red/green Opsin+ cone photoreceptor cells (**Fig. 2H** and data not shown). Immunostaining of Rhodopsin (a marker of mature rod photoreceptors) revealed no Rhodopsin+ cells at differentiation day 100 (data not shown). Rhodopsin+ cells appeared at differentiation day 120 with a stable efficiency of the three independent iPS cell lines from each patient (**Fig. 2K,N** **and Fig. S6C**). Additionally, 15.1±0.60% and 13.3±1.65% cells were positive for Recoverin (a conventional marker for both rod, cone photoreceptors and cone bipolar cells) in K21- and K11-iPS cells, respectively (data from three selected lines), consistent with stable differentiation. Furthermore, we confirmed rod induction by labeling with lentiviral vectors driving GFP from the Rhodopsin and Nrl promoters, either of which is specifically expressed in rod photoreceptors (**Fig. 2L–M**). Whole-cell patch-clamp recording demonstrated that the rod photoreceptor cell membrane contains voltage-dependent channels, suggesting that differentiated patient-derived rod cells are electrophysiologically functional (**Fig. 2N–O**). Meanwhile, the excluded iPS cell lines (ones that showed spontaneous differentiation during maintenance, or had a high copy number of transgenes), demonstrated a significant diversity of differentiation (**Fig. S7**). Together, these data show that patient-derived iPS cells can differentiate into cells that exhibit many of the immunochemical and electrophysiological features of mature rod photoreceptor cells.

### Patient-specific rod cells undergo degeneration *in vitro*

**Figure 3:**
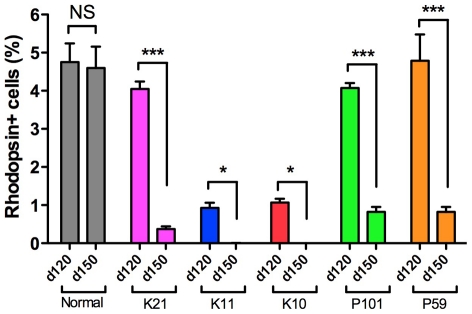
RP patient-derived rod photoreceptors undergo degeneration *in vitro*. iPS cells were differentiated into Rhodopsin+ rod photoreceptors in serum-free culture of embryoid body-like aggregates (SFEB culture). The percentages of Rhodopsin+ rod photoreceptors were evaluated at both day 120 and day 150, respectively. Data were from three independent iPS cell lines derived from the patients. ANOVA followed by Dunnett's test. * *p*<0.05; ***p<0.001. Values in the graphs are means and s.e.m.

As compared with normal iPS cells, there is no significant difference in rod cell differentiation efficiency at day 120 in K21(RP1)-, P101(PRPH2)-, and P59(RHO)-iPS cell lines (**Fig. 3**). iPS cells from both K11(RP9) and K10(RP9) carried a RP9 mutation; however, rod cell number was significantly lower than in normal iPS cells (**Fig. 3**). We asked whether early death of precursor cells leads to a smaller number of mature rod photoreceptor cells. To determine whether genetic mutations induce degeneration in photoreceptors cells *in vitro*, we extended the culture period and evaluated the number of rod photoreceptors at day 150. In differentiated iPS cells from patient K21(RP1) at day 150, the number of Rhodposin+ cells was significantly decreased (**Fig. 3**). For the K11-iPS cells, no Rhodposin+ cells were found at day 150 (**Fig. 3**). Importantly, some K11-cells at day 150 were positive for Recoverin (10.3±1.99%) and Crx, markers for the rod, cone photoreceptors, and/or bipolar cells (**Fig. 2K** and data not shown), strongly suggesting that cone photoreceptor and/or bipolar cells survived, whereas the rod photoreceptors underwent degeneration *in vitro*. In addition, we detected cells positive for Islet1 (a marker for retinal amacrine, bipolar and ganglion cells), again consistent with the survival of other types of retinal cells (**Fig. S6F**). From these results, we concluded that mature rod photoreceptors differentiated from patient iPS cells selectively degenerate in an RP-specific manner *in vitro*.

### Cellular stresses involved in patient-derived rod cells

**Figure 4:**
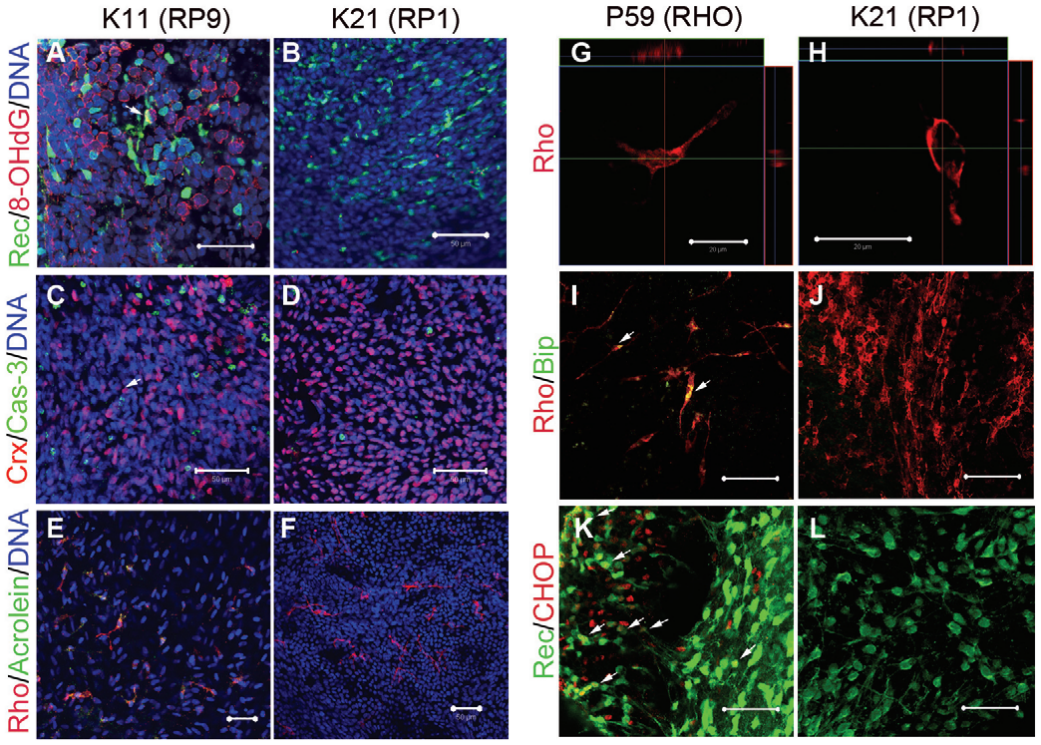
Cellular stress in patient-derived rod photoreceptor cells. Oxidative stress and apoptosis in differentiated rod photoreceptor cells derived from RP9-iPS (**A,C,E**) and RP1-iPS (**B,D,F**). (**A**) 8-OHdG, a marker for DNA oxidation, was found in K11- or K10-iPS−derived differentiated cells (day 100), but not in K21-iPS (**B**). Arrow indicates a cell double-positive for 8-OHdG and Recoverin. (**C**) The number of activated Caspase-3+ cells was greater in K11-iPS differentiation than in K21-iPS (**D**). From day 120, rod photoreceptor cells (Rhodopsin+) derived from RP9-iPS co-expressed the oxidative stress marker Acrolein (**E**); whereas RP1-iPS derivatives did not (**F**). (**G–L**) Abnormal cellular localization of Rhodopsin proteins and endoplasmic reticulum stress in RHO-iPS−derived rod photoreceptors. High magnification revealed cytoplasmic localization of Rhodopsin in rod photoreceptor cells carrying a RHO mutation (**G**) and a normal localization in the cell membrane in K21 cells (**H**). Rod cells derived from RHO-iPS co-expressed the ER stress markers BiP (**I**) and CHOP (**K**). K21-iPS−derived rod cells did not express BiP (**J**) or CHOP (**L**). Arrows indicate double-positive cells. Rec, Recoverin; Rho, Rhodopsin. All scale bars are 50 µm except for G and H (20 µm).

We next asked how the patient-derived rod photoreceptors degenerate. We evaluated apoptosis and cellular stresses in each cell line at both day 100 and day 120, respectively. Interestingly, in the RP9-iPS (K10 and K11) cells, a subset of Recoverin+ cells co-expressed cytoplasmic 8-hydroxy-2'-deoxyguanosine (8-OHdG), a major oxidative stress marker, indicating the presence of DNA oxidation in RP9 patient-derived photoreceptors by differentiation day 100 (**Fig. 4A** and **Fig. S8**). More caspase-3+ cells were presented in the Crx+ photoreceptor cluster of RP9-iPS than in those from other lines (**Fig. 4C–D**). After maturation of the rod photoreceptors from RP9-iPS cells, Rhodopsin+ cells co-expressed Acrolein, a marker of lipid oxidation (**Fig. 4E**), while no Rhodopsin+/Acrolein+ cells were observed in iPS cells derived from other patients carrying different mutations or in normal iPS cells (**Fig. 4F**). This pattern was similar to the cases of 8-OHdG and activated caspase-3. Thus, we conclude that oxidation is involved in the RP9-rod photoreceptor degeneration.

In differentiated RHO-iPS (P59) cells, we found that Rhodopsin proteins were localized in the cytoplasm (**Fig. 4G**), as determined by immunostaining with anti-Rhodopsin antibody (Ret-P1). This pattern is unlike the normal localization of Rhodopsin at the cell membrane in photoreceptors derived from normal iPS or other patient-derived iPS cells (**Fig. 4H** and data not shown). This result suggests accumulation of unfolded Rhodopsin, as reported previously in rhodopsin mutant mice cells [13]. We next examined the possible involvement of endoplasmic reticulum (ER) stress in RHO-iPS cell line degeneration. The Rhodopsin+ or Recoverin+ cells co-expressed immunoglobulin heavy-chain binding protein (BiP) or C/EBP homologous protein (CHOP), two conventional markers of endoplasmic reticulum (ER) stress, from day 120 (**Fig. 4I,K** **and Fig. S9**), while cells derived from control iPS or other mutant iPS cells were negative for BiP and CHOP (**Fig. 4J,L**). Taken together, these results demonstrate that ER stress is involved in rod photoreceptors carrying a RHO mutation.

### Drug evaluation in patient-specific rod cells

**Figure 5:**
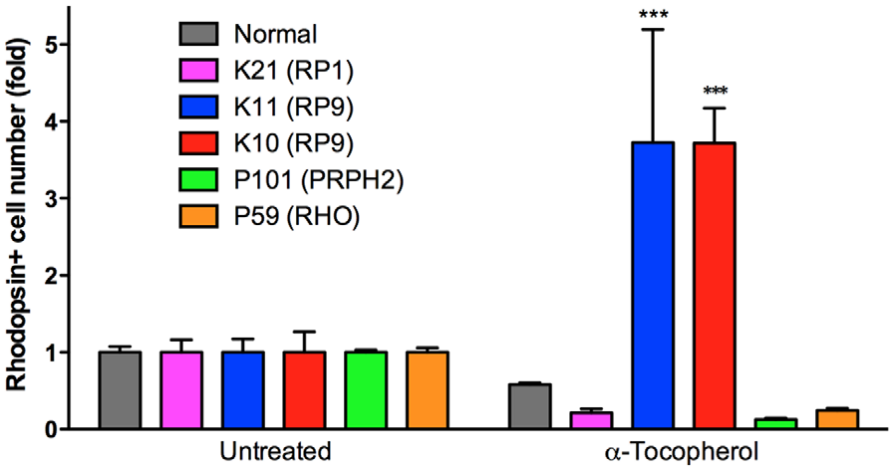
Disease modeling of patient-derived rod photoreceptor cells. α-Tocopherol treatment of patient-specific rod photoreceptors yielded a significant beneficial effect in RP9 mutant cells. Two-way ANOVA Bonferroni post-test showed no significance in other group (n = 3–8). Data represent 1–2 selected iPS cell lines of each patient. ***p<0.001. Values in the graphs are means and s.e.m.

The antioxidant vitamins α-tocopherol, ascorbic acid, and β-carotene have been tested in clinical trials as dietary therapies for RP [2] and in another major retinal degenerative disease, age-related macular degeneration [14]. Thus far, mostly due to the lack of appropriate validation models, there has been no evidence supporting the beneficial effects of these compounds on rod photoreceptors. We therefore assessed the effects of these agents on rod photoreceptors derived from patient iPS cells. In mouse retinal culture, short-term treatment with α-tocopherol, ascorbic acid and β-carotene at 100 µM, 200 µM and 1.6 µM, respectively, exerted no significant toxic effects on rod photoreceptor cells (**Fig. S10**). Since the differentiated rod photoreceptors underwent degeneration after day 120, we treated the cells for 7 days with these agents starting at day 120 (**Fig. 2A**). α-Tocopherol treatment significantly increased the number of Rhodopsin+ cells in iPS cells derived from K11- and K10-iPS with the RP9 mutation, while it had no significant effects on iPS cells with the either the RP1, PRPH2 or RHO mutation (**Fig. 5**). In contrast, neither ascorbic acid nor β-carotene treatment had any effect on iPS cells of any genotype (**Fig. S11**). We cannot currently explain the discrepancy between the effects of these antioxidants. It has been reported that under certain circumstances, anti-oxidants can act as “pro-oxidants” [15]. Taken together, our results indicate that treatment with α-tocopherol is beneficial to RP9-rod photoreceptor survival, and causes different effects on Rhodposin+ cells derived from different patients.

## Discussion

By using patient-derived iPS cells and *in vitro* differentiation technology, we have shown that RP9-retinitis pigmentosa is involved, at least in part, in oxidative stress pathways; this has not been reported previously in any animals or cell models. Furthermore, we have demonstrated that the antioxidant α-tocopherol exerts a beneficial effect on RP9-rod cells. Additionally, we have clearly shown that rod photoreceptors derived from patients with a RHO mutation are associated with ER stress; this is the first report of ER stress in a cell culture model for human rod cells. These cell models will be very useful for disease mechanism dissection and drug discovery. By screening several drugs that had already been tested in RP patients, we have revealed that rod photoreceptor cells derived from RP patients with different genetic subtypes exhibit significant differences in drug responses. Among the different types of antioxidants, α-tocopherol has either beneficial or non-beneficial effects on diseased photoreceptors, depending on the genetic mutation. This is the first report of the utilization of iPS cells related to personalized medicine, which will be helpful for routin clinical practice. Our results also provided evidence that genetic diagnosis is essential for optimizing personalized treatment for patients with retinal degenerative diseases [11]. An important future study made possible by this work is the screening of a compound library for drugs that could be used to treat RP. Patient-derived iPS cells revealed differences in pathogenesis and the efficacy of antioxidants among patients with different disease-causing mutations. Although the microenvironment affects the pathogenesis of diseases, and *in vitro* evaluation is not perfect, this study suggests that iPS cells could be used to select between multiple available treatments, allowing physicians to advise each patient individually. The weakness of our method for disease modeling is that differentiation requires a long period of time. Shortening the induction period and identifying appropriate surface markers for rod cells will improve disease modeling using patient-specific iPS cells.

In brief, we generated pluripotent stem cells from retinitis pigmentosa patients and induced them into retinal cells. Compared with normal cells, patient-derived rod cells simulated the disease phenotype and exhibited different responses to specific drugs. We found that patient-specific rod cells underwent degeneration *in vitro*, which maybe related to different cellular stresses. To our knowledge, this is the first report of disease modeling of retinal degeneration using patient-derived iPS cells.

## Supporting Information

Figure S1 Pedigrees of K21 (A), P59 (B), K10 and K11 (C). Families of P59 (**B**) and K10 and K11 (**C**) show autosomal dominant mode of inheritance. (**C**) Mutation analysis was performed in four patients and two normal relatives in the RP9 family. The H137L mutation in RP9 gene was co-segregated with the disease in the family. Closed symbols indicate individuals with RP and open symbols indicate unaffected subjects. Question marks indicate symptom unknown. The bars above the symbols indicate examined subjects. Arrow, proband; slash, deceased.
(TIF)

Figure S2 **Mutation in the** ***RP9*** **gene.** (**A**) Alignment of RP9 sequence and pseudo-gene shows the same nucleotide in the mutated location. (**B**) Sequence chromatogram of cDNA sequence demonstrates the c.410A>T (H137L) mutation in the *RP9* gene, instead of the paralogous variant in pseudo-gene which was documented in RetNet (www.sph.uth.tmc.edu/retnet/disease.htm).
(JPG)

Figure S3 **Selection by colony morphology.** (**A**) iPS colony (K21S4) shows ES-like morphology. (**B**) Spontaneous differentiation in the colony during maintenance (K21S14). Scale bars, 50 µm.
(TIF)

Figure S4 **Quantification of transgene copy number.** Total copy number of four transgenes in the selected iPS lines. Selected iPS cells with fewest integrations and two high copy number lines used for *in vitro* differentiation.
(TIF)

Figure S5 **Efficiency of RPE induction in patient-iPS cells.** RPE production of the five patient-iPS cells showed no significant differences (n = 4). Data represent the percentage of RPE area at differentiation day 60. One-way ANOVA followed by Dunnett's test. Values are mean and s.e.m.
(TIF)

Figure S6 **Induced retinal cells from patient iPS cells (K21S4).** Crx+ photoreceptor precursor cells present in the cell cluster on differentiation day 60 (A). Crx+ cells co-expressed Recoverin, indicating differentiation into photoreceptor cells (B). Rhodopsin+ cells had a long process at day 150 (C). In the differentiated cells, we also observed cells positive of PKCα (a marker for bipolar cells) (D). Cells positive for Math5 and Brn3b (markers for ganglion progenitor or ganglion cells (day 60) (E). Cells positive for Islet-1 (a marker for amacrine, bipolar and ganglion cells) (F). Scale bars, 50 µm (A, D, E, and F); 20 µm (B and C).
(TIF)

Figure S7 **Differentiation of the patient-iPS cells.** iPS colony was cut into uniform sized pieces (**A**) and subjected to a floating culture (P59M8, day 20) (**B**). RPE (pigmented) and recoverin+ (green) cells were efficiently induced (P59M8, day 60) (**C**). (**D**) An excluded iPS line, P59M16, with high number transgenes showed a striking lentoid formation during the floating culture (day 20). Scale bars, 50 µm.
(TIF)

Figure S8 **Oxidative stress in photoreceptor cells with the RP9 mutation (K11).** (A) Recoverin, (B) 8-OHdG, (C) Recoverin/8-OHdG, (D) Recoverin/8-OHdG/DNA. Arrows indicate cells with weak Recoverin signal positive for 8-OHdG; Arrowheads represent cells with strong Recoverin signal positive for 8-OHdG; Asterisks represent Recoverin+ cells negative for 8-OHdG. Scale bar, 50 µm.
(JPG)

Figure S9 **ER stress in photoreceptor cells with the RHO mutation (P59).** (A) CHOP, (B) Recoverin, (C) Recoverin/CHOP, (D) Recoverin/CHOP/DNA. Arrows indicate cells with weak Recoverin signals positive for CHOP in nuclei; Arrowheads represent cells with strong Recoverin signals positive for CHOP; Asterisks represent Recoverin+ cells negative for CHOP. Scale bar, 50 µm.
(JPG)

Figure S10 **Toxicity testing of the antioxidants in murine retina-derived rod photoreceptor cells.** Primary culture of mouse retinal cells treated with 100 µM α-tocopherol, 200 µM ascorbic acid or 1.6 µM β-carotene for 24 hours and the rod photoreceptors were counted using flow cytometry. Value represents the ratio of treated-rod photoreceptors compared with control cells. n = 4. One-way ANOVA followed by Dunnett's test. Values are mean and s.e.m. NS, not significant.
(JPG)

Figure S11 **Differentiated rod cells from normal and patient iPS cells treated with 200 µM ascorbic acid or 1.6 µM β-carotene did not show statistically significant differences.** Two-way ANOVA Bonferroni post-test. Values are mean and s.e.m.
(JPG)

Table S1 **Phenotypic data of the RP patients.** M, male; F, female; AD, age at diagnosis; BCVA, best corrected visual acuity; HM, hand motion.
(DOC)

Table S2 **Antibodies used in the present study.**
(DOC)

## References

[1] Weleber RG, Gregory-Evans K, Hilton DR, Schachat AP, Ryan SJ (2006). Retinitis Pigmentosa and Allied Disorders.. Retina. Elsevier Mosby.

[2] Berson EL, Rosner B, Sandberg MA, Hayes KC, Nicholson BW (1993). A randomized trial of vitamin A and vitamin E supplementation for retinitis pigmentosa.. Arch Ophthalmol.

[3] Takahashi K, Tanabe K, Ohnuki M, Narita M, Ichisaka T (2007). Induction of pluripotent stem cells from adult human fibroblasts by defined factors.. Cell.

[4] Yu J, Vodyanik MA, Smuga-Otto K, Antosiewicz-Bourget J, Frane JL (2007). Induced pluripotent stem cell lines derived from human somatic cells.. Science.

[5] Park IH, Arora N, Huo H, Maherali N, Ahfeldt T (2008). Disease-specific induced pluripotent stem cells.. Cell.

[6] Raya A, Rodríguez-Pizà I, Guenechea G, Vassena R, Navarro S (2009). Disease-corrected haematopoietic progenitors from Fanconi anaemia induced pluripotent stem cells.. Nature.

[7] Yamanaka S (2007). Strategies and new developments in the generation of patient-specific pluripotent stem cells.. Cell Stem Cell.

[8] Osakada F, Ikeda H, Mandai M, Wataya T, Watanabe K (2008). Toward the generation of rod and cone photoreceptors from mouse, monkey and human embryonic stem cells.. Nat Biotechnol.

[9] Osakada F, Jin ZB, Hirami Y, Ikeda H, Danjyo T (2009). In vitro differentiation of retinal cells from human pluripotent stem cells by small-molecule induction.. J Cell Sci.

[10] Hirami Y, Osakada F, Takahashi K, Okita K, Yamanaka S (2009). Generation of retinal cells from mouse and human induced pluripotent stem cells.. Neurosci Lett.

[11] Jin ZB, Mandai M, Yokota T, Higuchi K, Ohmori K (2008). Identifying pathogenic genetic background of simplex or multiplex retinitis pigmentosa patients: a large scale mutation screening study.. J Med Genet.

[12] Ikeda H, Osakada F, Watanabe K, Mizuseki K, Haraguchi T (2005). Generation of Rx+/Pax6+ neural retinal precursors from embryonic stem cells.. Proc Natl Acad Sci U S A.

[13] Sung CH, Davenport CM, Nathans J (1993). Rhodopsin mutations responsible for autosomal dominant retinitis pigmentosa. Clustering of functional classes along the polypeptide chain.. J Biol Chem.

[14] van Leeuwen R, Boekhoorn S, Vingerling JR, Witteman JC, Klaver CC (2005). Dietary intake of antioxidants and risk of age-related macular degeneration.. JAMA.

[15] van Helden YG, Keijer J, Heil SG, Picó C, Palou A (2009). Beta-carotene affects oxidative stress-related DNA damage in lung epithelial cells and in ferret lung.. Carcinogenesis.

