# Supplementary figures and images for: Modeling Retinal Degeneration Using Patient-Specific Induced Pluripotent Stem Cells
Source: PLoS One. 2011 Feb 10;6(2):e17084. doi: 10.1371/journal.pone.0017084 (PMC3037398; doi:10.1371/journal.pone.0017084)

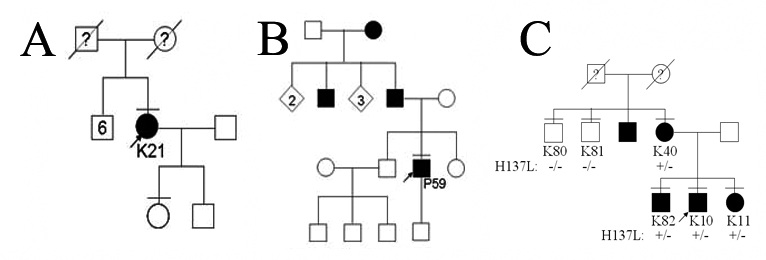

Supplement: Figure S1 — Pedigrees of K21 (A), P59 (B), K10 and K11 (C). Families of P59 (B) and K10 and K11 (C) show autosomal dominant mode of inheritance. (C) Mutation analysis was performed in four patients and two normal relatives in the RP9 family. The H137L mutation in RP9 gene was co-segregated with the disease in the family. Closed symbols indicate individuals with RP and open symbols indicate unaffected subjects. Question marks indicate symptom unknown. The bars above the symbols indicate examined subjects. Arrow, proband; slash, deceased. (TIF) [file pone.0017084.s001.tif]

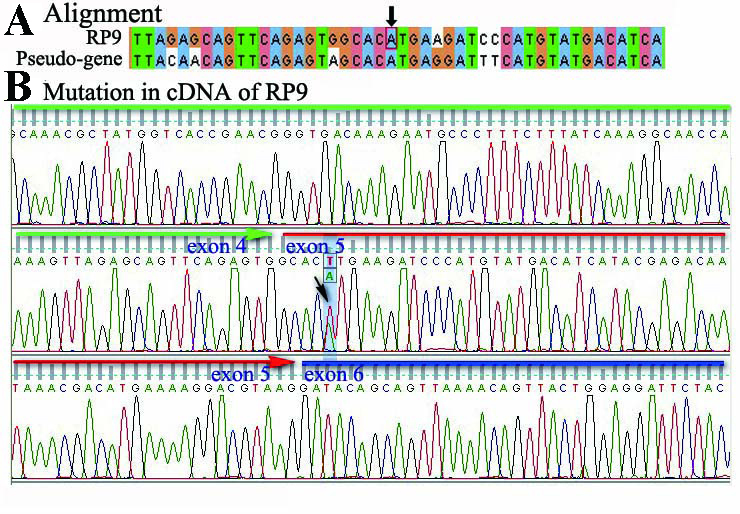

Supplement: Figure S2 — Mutation in the RP9 gene. (A) Alignment of RP9 sequence and pseudo-gene shows the same nucleotide in the mutated location. (B) Sequence chromatogram of cDNA sequence demonstrates the c.410A>T (H137L) mutation in the RP9 gene, instead of the paralogous variant in pseudo-gene which was documented in RetNet (www.sph.uth.tmc.edu/retnet/disease.htm). (JPG) [file pone.0017084.s002.jpg]

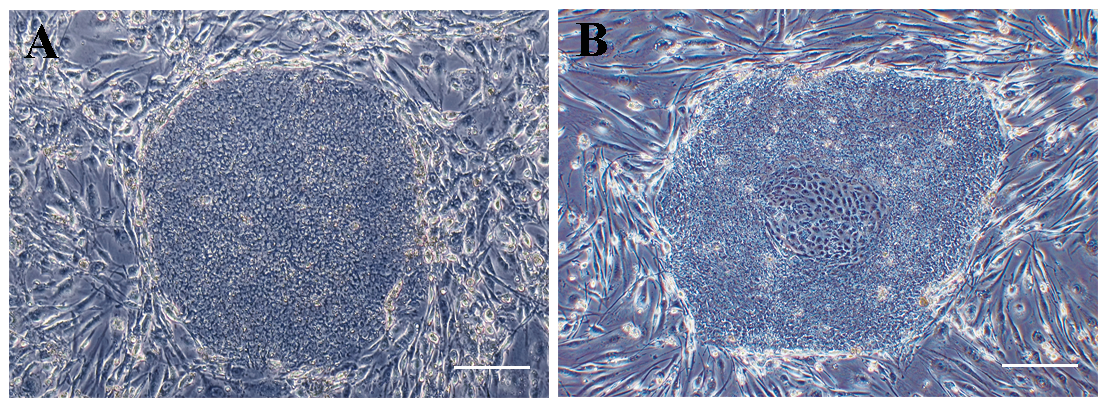

Supplement: Figure S3 — Selection by colony morphology. (A) iPS colony (K21S4) shows ES-like morphology. (B) Spontaneous differentiation in the colony during maintenance (K21S14). Scale bars, 50 µm. (TIF) [file pone.0017084.s003.tif]

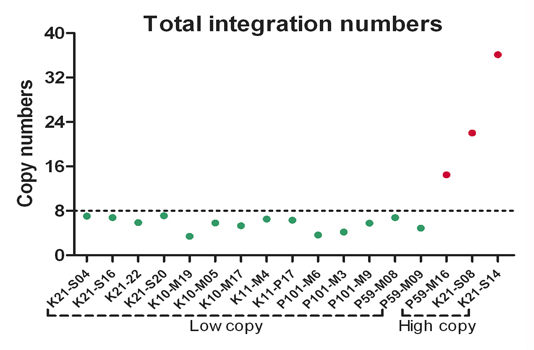

Supplement: Figure S4 — Quantification of transgene copy number. Total copy number of four transgenes in the selected iPS lines. Selected iPS cells with fewest integrations and two high copy number lines used for in vitro differentiation. (TIF) [file pone.0017084.s004.tif]

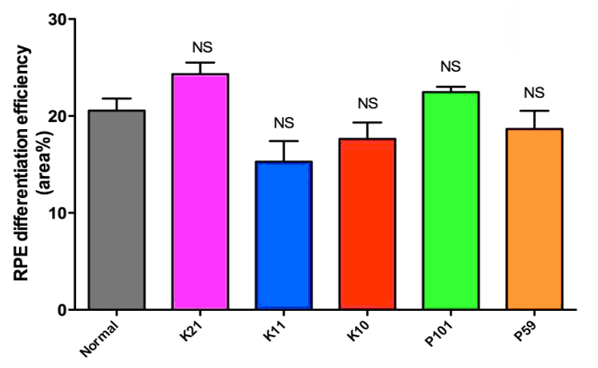

Supplement: Figure S5 — Efficiency of RPE induction in patient-iPS cells. RPE production of the five patient-iPS cells showed no significant differences (n = 4). Data represent the percentage of RPE area at differentiation day 60. One-way ANOVA followed by Dunnett's test. Values are mean and s.e.m. (TIF) [file pone.0017084.s005.tif]

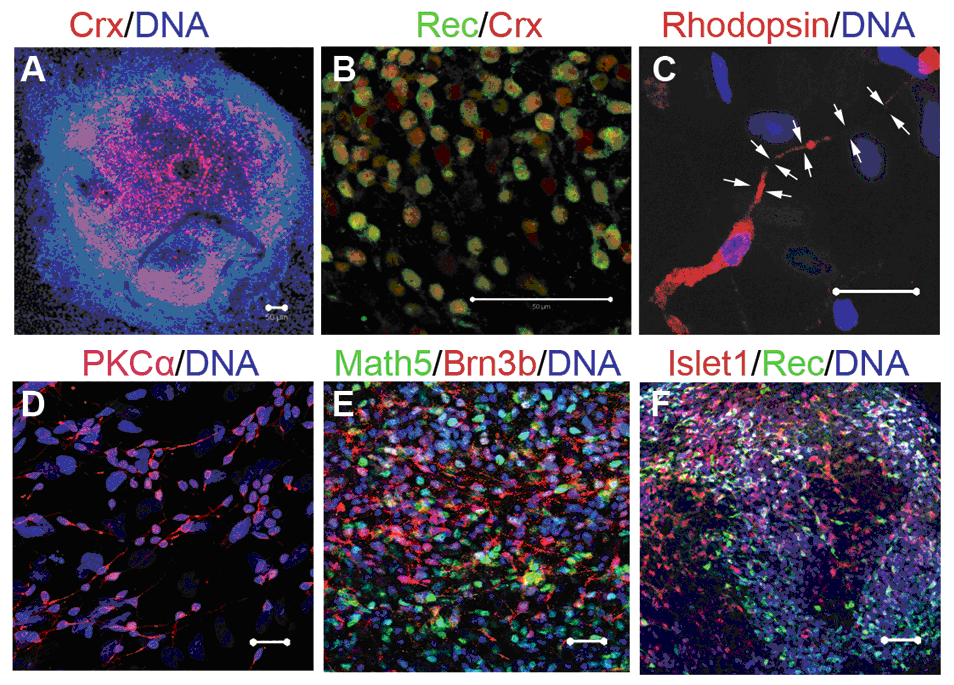

Supplement: Figure S6 — Induced retinal cells from patient iPS cells (K21S4). Crx+ photoreceptor precursor cells present in the cell cluster on differentiation day 60 (A). Crx+ cells co-expressed Recoverin, indicating differentiation into photoreceptor cells (B). Rhodopsin+ cells had a long process at day 150 (C). In the differentiated cells, we also observed cells positive of PKCα (a marker for bipolar cells) (D). Cells positive for Math5 and Brn3b (markers for ganglion progenitor or ganglion cells (day 60) (E). Cells positive for Islet-1 (a marker for amacrine, bipolar and ganglion cells) (F). Scale bars, 50 µm (A, D, E, and F); 20 µm (B and C). (TIF) [file pone.0017084.s006.tif]

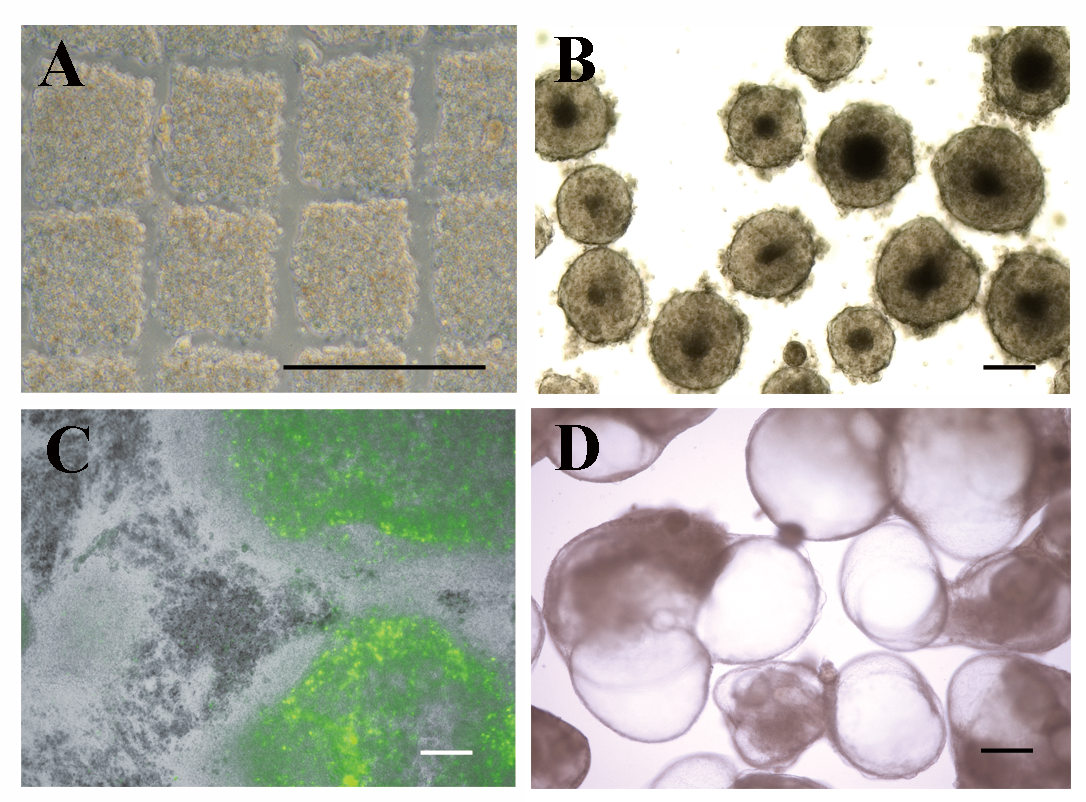

Supplement: Figure S7 — Differentiation of the patient-iPS cells. iPS colony was cut into uniform sized pieces (A) and subjected to a floating culture (P59M8, day 20) (B). RPE (pigmented) and recoverin+ (green) cells were efficiently induced (P59M8, day 60) (C). (D) An excluded iPS line, P59M16, with high number transgenes showed a striking lentoid formation during the floating culture (day 20). Scale bars, 50 µm. (TIF) [file pone.0017084.s007.tif]

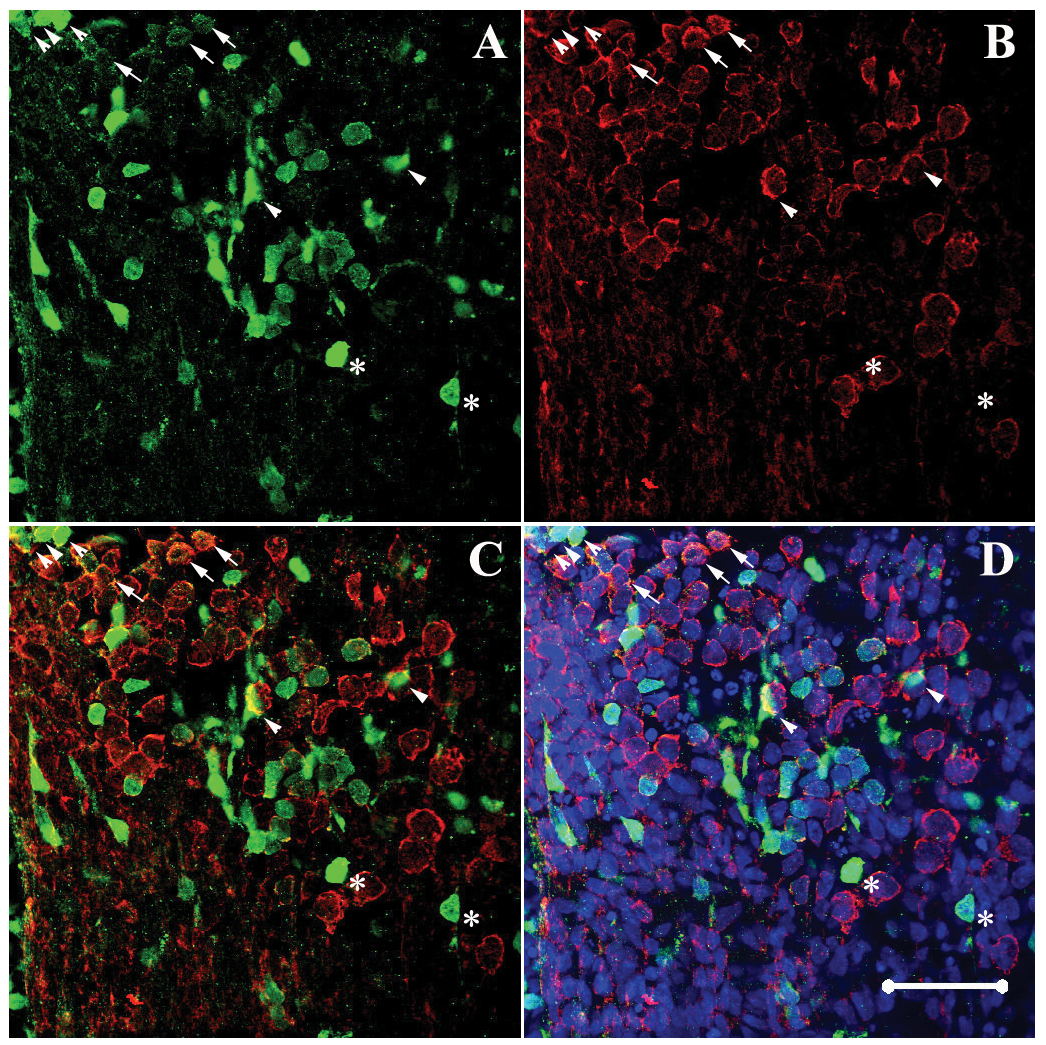

Supplement: Figure S8 — Oxidative stress in photoreceptor cells with the RP9 mutation (K11). (A) Recoverin, (B) 8-OHdG, (C) Recoverin/8-OHdG, (D) Recoverin/8-OHdG/DNA. Arrows indicate cells with weak Recoverin signal positive for 8-OHdG; Arrowheads represent cells with strong Recoverin signal positive for 8-OHdG; Asterisks represent Recoverin+ cells negative for 8-OHdG. Scale bar, 50 µm. (JPG) [file pone.0017084.s008.jpg]

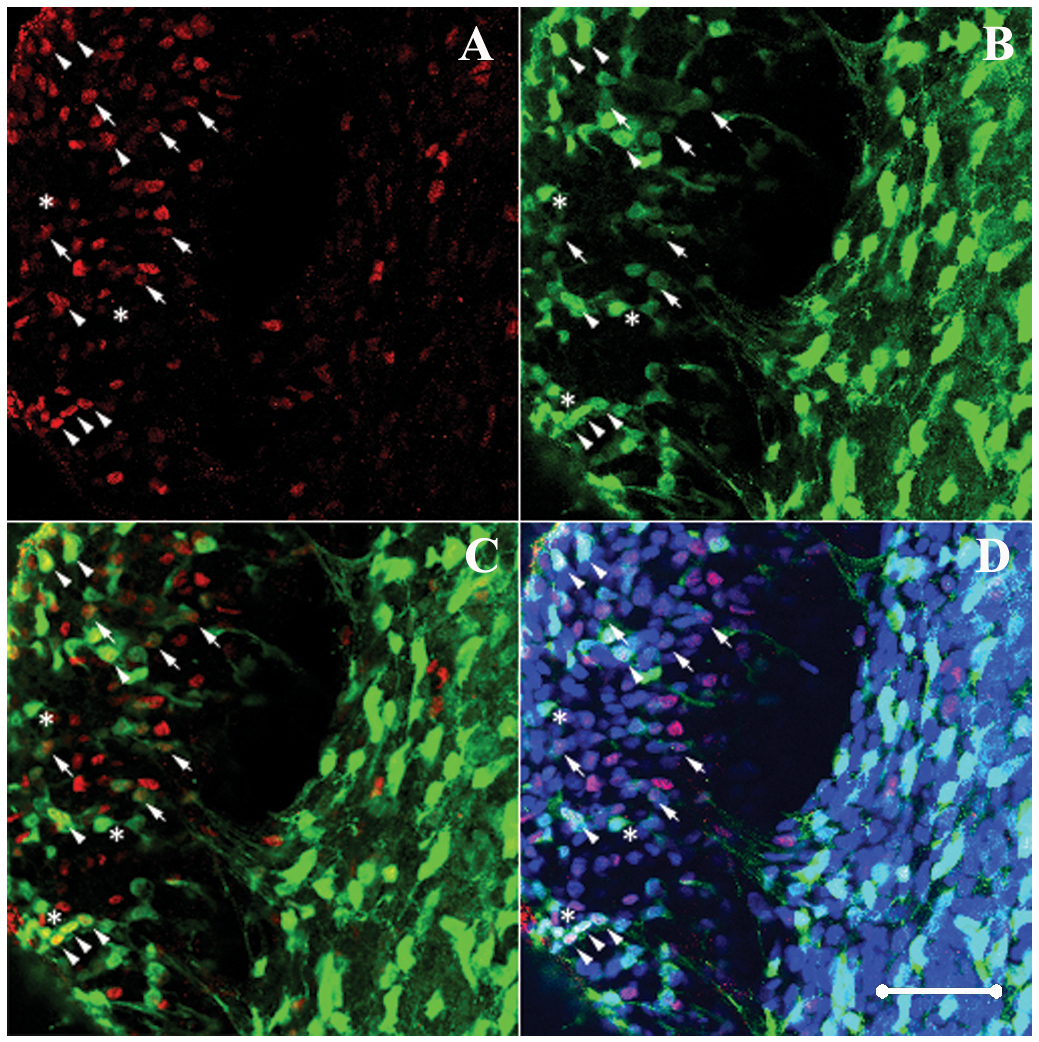

Supplement: Figure S9 — ER stress in photoreceptor cells with the RHO mutation (P59). (A) CHOP, (B) Recoverin, (C) Recoverin/CHOP, (D) Recoverin/CHOP/DNA. Arrows indicate cells with weak Recoverin signals positive for CHOP in nuclei; Arrowheads represent cells with strong Recoverin signals positive for CHOP; Asterisks represent Recoverin+ cells negative for CHOP. Scale bar, 50 µm. (JPG) [file pone.0017084.s009.jpg]

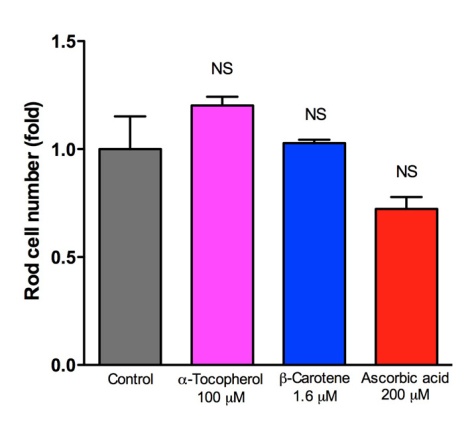

Supplement: Figure S10 — Toxicity testing of the antioxidants in murine retina-derived rod photoreceptor cells. Primary culture of mouse retinal cells treated with 100 µM α-tocopherol, 200 µM ascorbic acid or 1.6 µM β-carotene for 24 hours and the rod photoreceptors were counted using flow cytometry. Value represents the ratio of treated-rod photoreceptors compared with control cells. n = 4. One-way ANOVA followed by Dunnett's test. Values are mean and s.e.m. NS, not significant. (JPG) [file pone.0017084.s010.jpg]

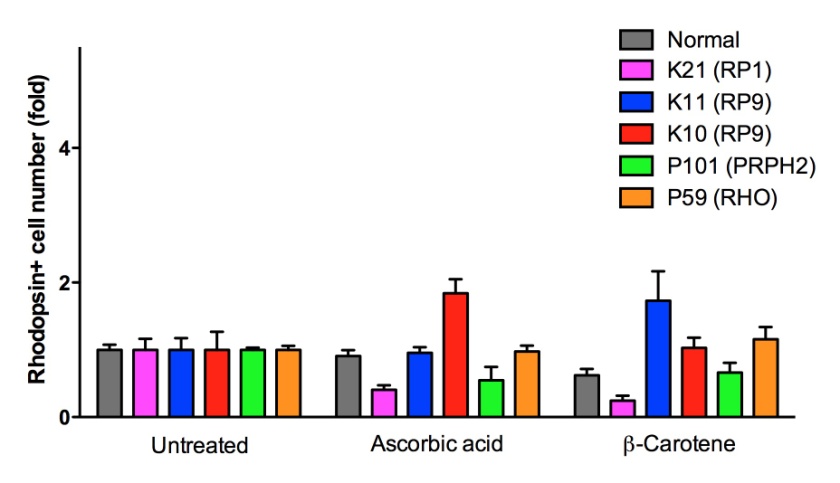

Supplement: Figure S11 — Differentiated rod cells from normal and patient iPS cells treated with 200 µM ascorbic acid or 1.6 µM β-carotene did not show statistically significant differences. Two-way ANOVA Bonferroni post-test. Values are mean and s.e.m. (JPG) [file pone.0017084.s011.jpg]
