# Supplementary material for: Modeling Retinal Degeneration Using Patient-Specific Induced Pluripotent Stem Cells
Source: PLoS One. 2011 Feb 10;6(2):e17084. doi: 10.1371/journal.pone.0017084 (PMC3037398; doi:10.1371/journal.pone.0017084)
Supplement: Table S1 — Phenotypic data of the RP patients. M, male; F, female; AD, age at diagnosis; BCVA, best corrected visual acuity; HM, hand motion. (DOC) [file pone.0017084.s012.doc]

**Table S**1. Phenotypic data of the RP patients

| Case | M/F | Age  (year) | AD (year) | BCVA | | Fundoscopic examination |
| --- | --- | --- | --- | --- | --- | --- |
|  |  |  |  | Right eye | Left eye |  |
| K21 | F | 67 | 40 | HM/20cm | HM/20cm | Pigmentation, arteriolar attenuation |
| K11 | F | 42 | 10 | HM/5cm | HM/30cm | Pigmentation, arteriolar attenuation |
| K10 | M | 39 | 12 | 0.2 | 0.15 | Pigmentation, arteriolar attenuation |
| P101 | F | 67 | 47 | 0.3 | 0.3 | Pigmentation, arteriolar attenuation |
| P59 | M | 40 | 39 | 0.6 | 0.6 | Pigmentation, arteriolar attenuation |
