# Supplementary material for: Modeling Retinal Degeneration Using Patient-Specific Induced Pluripotent Stem Cells
Source: PLoS One. 2011 Feb 10;6(2):e17084. doi: 10.1371/journal.pone.0017084 (PMC3037398; doi:10.1371/journal.pone.0017084)
Supplement: Table S2 — Antibodies used in the present study. (DOC) [file pone.0017084.s013.doc]

**Table S2. Antibodies used in the present study.**

| **Antibody** | **Host Specie** | **Source** | **Dilution** |
| --- | --- | --- | --- |
| Oct3/4 | Mouse | BD Pharmingen | 1:200 |
| Nanog | Rabbit | ReproCELL | 1:1000 |
| SSEA-3 | Rat | Millipore | 1:200 |
| Tra-1-60 | Mouse | Millipore | 1:300 |
| Pax6 | Rabbit | Covance | 1:600 |
| Pax6 | Mouse | DSHB | 1:200 |
| Rx | Rabbit | Provided by Y. Sasai | 1:200 |
| Mitf | Mouse | Abcam | 1:30 |
| Crx | Rat | Provided by Y. Sasai | 1:200 |
| Rhodopsin (RET-P1) | Mouse | Sigma | 1:2000 |
| Recoverin | Rabbit | Millipore | 1:3000 |
| Ki67 | Mouse | BD Pharmingen | 1:200 |
| Red/green Opsin | Rabbit | Millipore | 1:500 |
| Blue Opsin | Rabbit | Millipore | 1:500 |
| ZO-1 | Rabbit | Zymed | 1:100 |
| Islet-1 | Mouse | DSHB | 1:200 |
| PKCα | Rabbit | Sigma | 1:1000 |
| Math5 | Rabbit | Millipore | 1:1000 |
| Brn3b | Mouse | Millipore | 1:600 |
| Activated caspase-3 | Rabbit | BD Pharmingen | 1:500 |
| 8-OHdG | Mouse | JaICA | 1:10 |
| Acrolein | Rabbit | Cell Sciences | 1:500 |
| Bip | Rabbit | CST | 1:500 |
| CHOP | Mouse | CST | 1:1600 |
| Mouse IgG-cy2 or cy3 | Donkey | Jackson | 1:300 |
| Rat IgG-cy2 or cy3 | Donkey | Jackson | 1:300 |
| Rabbit IgG-cy2 or cy3 | Donkey | Jackson | 1:300 |
